# Intracellular Neuroprotective Mechanisms in Neuron-Glial Networks Mediated by Glial Cell Line-Derived Neurotrophic Factor

**DOI:** 10.1155/2019/1036907

**Published:** 2019-11-18

**Authors:** Еlena V. Mitroshina, Tatiana A. Mishchenko, Olesya M. Shirokova, Tatiana A. Astrakhanova, Maria M. Loginova, Ekaterina A. Epifanova, Alexey A. Babaev, Victor S. Tarabykin, Maria V. Vedunova

**Affiliations:** ^1^Lobachevsky State University of Nizhni Novgorod, 23 Prospekt Gagarina, Nizhny Novgorod 603950, Russia; ^2^Privolzhskiy Research Medical University, 10/1 Minin and Pozharsky Square, Nizhny Novgorod 603005, Russia; ^3^Institute of Cell Biology and Neurobiology, Charité-Universitätsmedizin Berlin, Charitéplatz 1, 10117 Berlin, Germany

## Abstract

Glial cell line-derived neurotrophic factor (GDNF) has a pronounced neuroprotective effect in various nervous system pathologies, including ischaemic brain damage and neurodegenerative diseases. In this work, we studied the effect of GDNF on the ultrastructure and functional activity of neuron-glial networks during acute hypoxic exposure, a key damaging factor in numerous brain pathologies. We analysed the molecular mechanisms most likely involved in the positive effects of GDNF. Hypoxia modelling was performed on day 14 of culturing primary hippocampal cells obtained from mouse embryos (E18). GDNF (1 ng/ml) was added to the culture medium 20 min before oxygen deprivation. Acute hypoxia-induced irreversible changes in the ultrastructure of neurons and astrocytes led to the loss of functional Сa^2+^ activity and neural network disruption. Destructive changes in the mitochondrial apparatus and its functional activity characterized by an increase in the basal oxygen consumption rate and respiratory chain complex II activity during decreased stimulated respiration intensity were observed 24 hours after hypoxic injury. At a concentration of 1 ng/ml, GDNF maintained the functional metabolic network activity in primary hippocampal cultures and preserved the structure of the synaptic apparatus and number of mature chemical synapses, confirming its neuroprotective effect. GDNF maintained the normal structure of mitochondria in neuronal outgrowth but not in the soma. Analysis of the possible GDNF mechanism revealed that RET kinase, a component of the receptor complex, and the PI3K/Akt pathway are crucial for the neuroprotective effect of GDNF. The current study also revealed the role of GDNF in the regulation of HIF-1*α* transcription factor expression under hypoxic conditions.

## 1. Introduction

Glial cell line-derived neurotrophic factor (GDNF) is known for its neurorestorative and neuroprotective effects in various pathologies, including Parkinson's disease [1–3], Alzheimer's disease [4, 5], and ischaemic damage [5–8], to the central and peripheral nervous systems. Despite numerous studies confirming the neuroprotective effect of GDNF, some preclinical and clinical data suggest that increasing GDNF concentrations do not always lead to significant long-term improvements [9, 10]. The mechanisms activated by GDNF injection could be more complex and associated with modification of numerous subcellular cascades in both neurons and astrocytes [11, 12]. Activation of these molecular reactions unites the neuron-glial network into a single functional and metabolic system capable of a comprehensive adaptive response [12, 13]. The main action of GDNF is associated with activation of the GFR*α*-mediated RET kinase metabolic cascade and its numerous alterations for cell activity correction [12, 14]. Studies on key intracellular kinases involved in the neuroprotective effect of GDNF allow for elucidation of crucial points of GDNF action and the mechanisms underlying its reduced effectiveness in certain conditions.

Hypoxia, as a key damaging factor in various brain pathologies [15–19], is of special interest. In the nervous system, hypoxia is a crucial factor because of the high level of neuronal metabolism, which is associated with substantial oxygen consumption. Although the adult brain mass accounts for only 2.5% of the body mass, the brain consumes up to 25% of the total oxygen absorbed by the body. Decreasing the oxygen concentration in nervous tissue leads to a catastrophic decline in neuron-glial network function effectiveness and inevitably results in dissociation of oxidative phosphorylation, impairment of mitochondrial membrane permeability and activation of free radical processes [13, 20–23].

Our previously obtained data revealed that GDNF (1 ng/ml) has pronounced neuroprotective properties, including its ability to maintain the viability of dissociated hippocampal cells and to preserve spontaneous bioelectrical activity during the posthypoxic period [13]. The analysis of the activation patterns and burst structure revealed the reorganization of network bursts under hypoxic conditions, which were influenced by GDNF. GDNF promoted the preservation of the neural network activation pattern and functional structure, but the excitation transmission time between electrodes was significantly higher than the control values [13].

In the current work, we focused on studying the molecular and cellular mechanisms by which GDNF maintains the viability and functional activity of neuron-glial networks in the posthypoxic period. Special attention was paid to determining the influence of GDNF on the ultrastructure and functional activity of mitochondria since these organelles are the first and most important link in the development of the hypoxic damaging effect. We demonstrated that pretreatment with GDNF promoted the preservation of the synaptic apparatus structure and the number of mature chemical synapses and maintained the normal morphology of mitochondria in neuronal outgrowth. We also considered features of functional Ca^2+^ activity in neuron-glial networks during the posthypoxic period. Ca^2+^ ions are key regulators of many metabolic processes and directly participate in synaptic transmission, and their detection in the cellular cytoplasm allows for detailed analysis of nerve cell activity. Using the Ca^2+^ imaging technique, we showed that pretreatment with GDNF partially preserved the metabolic functional activity of neuron-glial networks in the posthypoxic period. To determine the molecular mechanisms of GDNF action, we studied the key kinase-mediated cascades activated by the GDNF receptor complex RET-GFR*α* and the possible regulatory influence of GDNF on hypoxia-inducible factor 1-alpha (HIF-1*α*). We demonstrated a key role of the PI3K/Akt pathway in the implementation of GDNF neuroprotective effects as well as neurotrophic factor participation in the regulation of HIF-*α* expression in nervous cells.

## 2. Materials and Methods

### 2.1. Ethics Statement

All experimental protocols used in this study were approved by the Bioethics Committee of Lobachevsky University and carried out in accordance with Act708n (23 08 2010) of the Russian Federation National Ministry of Public Health, which states the rules of laboratory practice for the care and use of laboratory animals, and the Council Directive 2010/63 EU of the European Parliament (September 22, 2010) on the protection of animals used for scientific purposes. Pregnant C57BL/6J female mice were used in accordance with the following experiments: 8 animals for cell viability detection, 8 animals for Ca^2+^ activity recordings, 6 animals for electron microscopy studies, and 10 animals for registration of mitochondrial functional activity and real-time PCR analysis. The mice were killed by cervical vertebrae dislocation, and their embryos were then surgically removed and sacrificed by decapitation on day 18 of embryo gestation. Embryonic brains were then used for primary hippocampal culture preparation.

### 2.2. Cell Culture

Primary neuronal cells were obtained from mouse embryonic hippocampal tissue and cultivated on coverslips pretreated with polyethyleneimine solution (1 mg/ml) (Sigma-Aldrich, P3143, Germany) in accordance with protocols described in [24, 25]. In brief, surgically isolated hippocampi underwent 20 min of enzymatic treatment with 0.25% trypsin-ethylenediaminetetraacetic acid (EDTA, Invitrogen, 25200-056, United States). The obtained cell suspension was centrifuged at 1,000 rotations per min (rpm) for 3 min. Then, the supernatant was carefully removed, and the cell pellet was resuspended in culture medium Neurobasal™ medium (Invitrogen, 21103-049), 2% B27 (Invitrogen, 17504-044), 0.5 mM L-glutamine (Invitrogen, 25030-024), and 5% foetal bovine serum (PanEco, K055, Russia) and placed on substrates for cultivation at an approximate initial density of 9,000 cells/mm^2^. After 24 hours and every third day, 50% of the medium was replaced with medium containing 0.4% foetal bovine serum. The cultures (total of 186) were maintained under constant conditions of 35.5°C (5% CO_2_) and a humidified atmosphere in a cell culture incubator (Sanyo, Japan).

### 2.3. Hypoxia Model

Acute normobaric hypoxia was modeled on day 14 of culture development *in vitro* (DIV) by replacing the normoxic culture medium with a medium containing a low oxygen concentration for 10 min. The oxygen was displaced from the medium in a sealed chamber in which the air was replaced with an inert gas (argon). The oxygen concentration decreased from 3.26 ml/l (normoxia) to 0.37 ml/l (hypoxia) [13, 26].

GDNF (1 ng/ml, Millipore, GF030, USA) and kinase inhibitors (1 *μ*M) were added to the culture medium 20 min before hypoxia. The RET kinase inhibitor OICR0008751A01; RAF kinase inhibitor L-779450; MAP2K1/2, Erk2, and RAF kinase inhibitor RO-5126766; AKT1 kinase inhibitor 10-DEBC hydrochloride; and Jak1/Jak2 kinase inhibitor filgotinib were kindly provided by the laboratory of David Kaplan (Charite University, Berlin, Germany). In the “Hypoxia” group, hypoxia was induced without additional treatment.

### 2.4. Cell Viability Detection

The viability of primary hippocampal cells was estimated according to [26] using a Leica DMIL HC inverted fluorescence microscope (Leica, Germany) and the specific fluorescent dyes propidium iodide (Sigma-Aldrich, США) and bisbenzimide (Sigma-Aldrich, США) at concentrations of 5 *μ*g/ml and 1 *μ*g/ml, respectively. The cells were observed under a Leica DMIL HC inverted fluorescence microscope (Leica, Germany) with a 10x/0.2Ph1 objective. The ratio of the number of dead cells stained with propidium iodide to the total number of cells stained with bisbenzimide was calculated.

### 2.5. Ca^2+^ Imaging

Calcium events were detected by a specific calcium-sensitive dye, Oregon Green 488 BAPTA-1 АМ (OGB1, 0.4 *μ*M, Invitrogen, USA), dissolved in dimethylsulfoxide (DMSO) (Sigma-Aldrich, D8418) supplemented with 4% pluronic F-127 (Invitrogen, P-3000 MP) on a Zeiss LSM 510 confocal laser scanning microscope (Carl Zeiss, Germany) with a W Plan-Apochromat 20x/1.0 objective. OGB-1 fluorescence was excited by a 488 nm Argon laser, and the emission was detected with a 500–530 nm filter. A time series of 256 × 256 pixel images capturing 420 *μ*m × 420 *μ*m fields of view was recorded at 4 Hz. A confocal pinhole of 1 airy unit was used to obtain an axial optical slice resolution of 1.6 *μ*m.

Quantitative evaluation of Ca^2+^ transients was performed offline using custom-made software in C++ Builder according to [24, 27]. The following main parameters of the functional calcium activity in the primary cultures were analysed: duration of the calcium oscillations (time from the beginning to the end of an oscillation (s)), frequency of the calcium oscillations (average number of oscillations per min), and percentage of working cells (ratio of the number of cells in which at least one oscillation was recorded to the total number of cells (%)). Data analysis was performed using the following algorithm. First, on the obtained confocal images of primary neuronal cultures, the areas coinciding with the cell bodies were selected. The Ca^2+^ fluorescence for each cell in each frame was calculated as the average fluorescence intensity (*F*, relative units from 0 to 255) of all pixels in the selected area. To detect single Ca^2+^ signals, each trace from all of the cells was filtered by averaging two neighbouring points in the sample set. Next, a simple derivative of the signal was calculated by determining the difference between pairs of subsequent points. The oscillations were found from the derivative of the trace using a threshold detection algorithm. The threshold was estimated as the detection accuracy coefficient multiplied by the standard deviation of the derivative of the trace. Suprathreshold points on the derivative of the trace were taken as the beginnings and endings of the oscillations. The detection accuracy coefficient was empirically set to 0.45 [25, 27].

### 2.6. Electron Microscopy

The day after acute hypoxia modelling, the primary hippocampal cultures were fixed in 2.5% glutaraldehyde (Acros Organics, AC119980010, United States) and subsequently prepared for electron microscopy analysis according to the protocol described in [24].

Ultrathin sections prepared using a Leica EM UC7 ULTRA ultramicrotome (Leica, Germany) were stained with 4% uranyl acetate (SPI-chem, 02624-AB, United States), lead citrate, and trihydrate (SPI-chem, 512-26-5) and then examined with a Morgagni 268D transmission electron microscope (FEI Company, United States).

### 2.7. Registration of Mitochondrial Functional Activity

Primary hippocampal cells underwent enzymatic treatment with a versine-trypsin (3 : 1) solution and were then removed from the cultivated substrate 24 hours after hypoxia modelling. Mitochondria were isolated from hippocampal cells using the standard differential centrifugation method [28, 29]. The cells were placed in ice-cold isolation medium (70 mM saccharose, 210 mM mannitol, 30 mM HEPES, and 0.1 mM EDTA (pH 7.4)) and subjected to homogenization in a glass homogenizer. The obtained homogenate was centrifuged at 4,000 rpm for 10 min at 0°C, and the precipitate was resuspended in an incubation medium containing 210 mM mannitol, 70 mM saccharose, 0.1 mM EGTA, and 10 mM HEPES (pH 7.4). Quantitative analysis of the proteins in the isolated mitochondria was performed according to the Bradford method [24].

Oxygen consumption by the isolated mitochondria was registered polarographically using the high-resolution respirometer OROBOROS Oxygraph-2k (OROBOROS Instruments, Austria). The oxygen consumption rate was fixed using DatLab5 software (OROBOROS Instruments, Austria) and expressed in pmol/s/1 mg mitochondrial protein.

The state of the mitochondrial respiratory chain was evaluated according to the following parameters: the rate of oxygen consumption by mitochondria with a high substrate content, 5 mM glutamate and 5 mM malate (substrates of complex I), in the incubation medium (V4 state); the oxidative phosphorylation rate of the respiratory chain in the presence of 5 mM adenosine diphosphate (ADP) (V3 state); and the work intensity of the respiratory chain after the stimulation of complex II with 10 mM sodium succinate (V4 state) [24]. The ratio of the V3 and V4 states (respiratory control index) characterizing the degree of mitochondrial respiratory chain coupling was also analysed.

### 2.8. Real-Time Polymerase Chain Reaction (Real-Time PCR)

Quantitative real-time PCR was used to analyse the levels of GFR*α* receptor (Gfra1 gene) and HIF-1*α* (Hif1) expression. Total RNA was isolated from primary hippocampal cell cultures 24 hours after hypoxia exposure (15 DIV) using an ExtractRNA kit (Eurogen, Russia). Then, сDNA was synthesized by Moloney murine leukaemia virus (MMLV), reverse transcriptase (Eurogen, Russia), and a random primer.

Quantitative real-time PCR was performed with qPCR mix-HS SYBR (Eurogen, Russia) and the Applied Biosystems 7500 RT-PCR thermal cycler. The following primers were used: Gria2_fw3—5′-AGCCAAGGACTCGGGAAGTAAGG-3′; Gria2_rv3—5′-CACCAGCATTGCCAAACCAAGG-3′; Hif1a_fw1—5′-GCAATTCTCCAAGCCCTCCAAG-3′; Hif1a_rv1—5′-TTCATCAGTGGTGGCAGTTGTG-3′; Oaz1_fw—5′-AAGGACAGTTTTGCAGCTCTCC-3′; and Oaz1_rv—5′-TCTGTCCTCACGGTTCTTGGG-3′.

Data processing was carried out using the *ΔΔ*Ct method and a reference sample in which the target gene level was taken as a unit. Normalization was performed relative to the reference gene (Oaz1).

### 2.9. Statistical Analysis

All quantified data are presented as the mean ± standard error of the mean (SEM). Statistical analyses were performed using one-way ANOVA implemented in Sigma Plot 11.0 software (Systat Software, Inc.). The Dunnett post hoc test was used as a post hoc test following ANOVA. At least three independent biological repetitions were included for all experiments. Differences between groups were considered significant if the corresponding *p* value was less than 0.05.

## 3. Results

First, we analysed the influence of GDNF on cell viability and ultrastructural changes in primary hippocampal cultures, revealing a neuroprotective effect. Pretreatment with GDNF decreased the number of dead cells in culture (“Sham” 6.32 ± 2.26%, “Hypoxia” 49.55 ± 3.72%, “Hypoxia+GDNF” 11.12 ± 3.21%; ^∗^versus “Sham”; ^#^versus “Hypoxia”; *p* < 0.05, ANOVA, *N* = 9).

**Figure 1:**
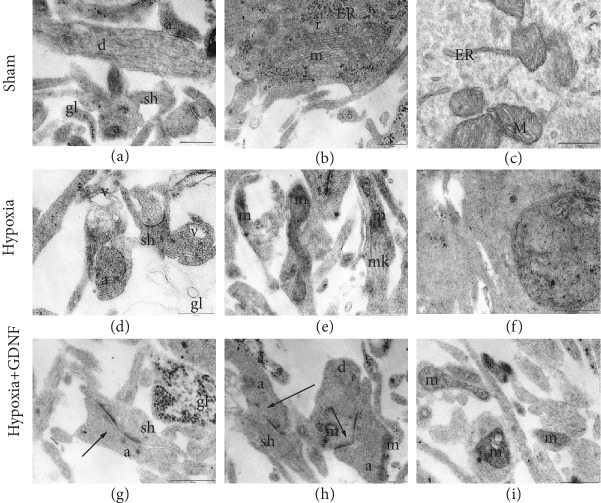
Representative electron microscopy images of dissociated hippocampal cells on the day after acute normobaric hypoxia modelling. (а–c) Sham, (d–f) Hypoxia, and (g–i) Hypoxia+GDNF. (а) Axo-spiny and axo-dendritic asymmetric synapses. Mitochondria in the postsynaptic terminal of an intact structure and vesicles in an axonal bud have equal size and osmiophility, and glial outgrowths are filled with osmiophilic granules. (b) Axo-dendritic synapse. Mitochondria have moderate osmiophility, many cristae, and ribosomes, including in the endoplasmic reticulum. (c) Mitochondria in a cell body and ribosomes, including in the endoplasmic reticulum. (d) Axo-spiny asymmetric contacts with a concave surface, vacuoles from destroyed mitochondria in the outgrowth, and a shell from the empty glial outgrowth. (e) Mitochondria in a neuronal outgrowth with an irregular form and osmiophilic vesicles with additional membranes among synaptic vesicles are visible in a single axon. (f) Impaired mitochondrion in a cell body; the internal structure is completely disrupted. (g) Axo-spiny asymmetric perforated contact and glial outgrowth with osmiophilic granules. (h) Axo-spiny and axo-dendritic perforated contacts and mitochondria in the axon have an irregular form. (i) Mitochondria with modified structures in different outgrowths. а: axon; v: vacuoles; gl: glial outgrowth; ER: granular endoplasmic reticulum; d: dendrite; m: mitochondria; r: ribosomes; sh: spine; black arrow: mature chemical synapse. Scale bar: 0.5 *μ*m.

Studies on the structures of mitochondria and the synaptic apparatus in the hypoxic state and GDNF influence are of special interest. Our recent data showed the formation of mature chemical synapses with a predominance of axo-dendritic and axo-spiny contacts on DIV 14. The number of gap junctions was lower at DIV 14 than at earlier stages of cultivation [30]. Mitochondrial cristae were well visualized (Figures 1(b) and 1(c)). The main pool of synaptic contacts was represented by vesicular symmetric and asymmetric axo-spiny and axo-dendritic contacts (Figure 1(a)). Mature chemical synapses with high postsynaptic density (PSD) and high osmiophility as well as various “weak” contacts with low osmiophilic PSD were observed, which are typical features of developing processes in neuron-glial networks.

Hypoxia primarily led to a decrease in the percentage of weak synapses (“Sham” 42.3 ± 4.19%, “Hypoxia” 26.01 ± 3.61%), while pretreatment with GDNF did not prevent a decrease in the percentage of osmiophilic synapses (“Hypoxia+GDNF” 26.31 ± 4.03%).

**Figure 2:**
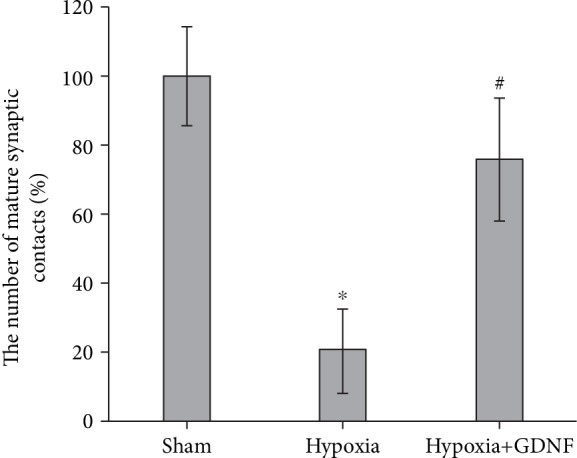
The number of mature synaptic contacts one day after pretreatment with GDNF (1 ng/ml) and acute normobaric hypoxia modelling *in vitro*. The data represent the mean values ± SEMs from three independent experiments. Statistical significance was calculated by one-way ANOVA; *p* < 0.05; ^∗^versus “Sham”; ^#^versus “Hypoxia”.

Moreover, in the “Hypoxia+GDNF” group, the number of mature synaptic contacts/100 *μ*m^2^ did not differ from that in the “Sham” group, whereas in the “Hypoxia” group, this parameter was significantly decreased (“Sham” 498.6 ± 87.7; “Hypoxia” 101.34 ± 48.7^∗^, “Hypoxia+GDNF” 378.9 ± 65.4, *p* < 0.05, ANOVA, *N* = 3). The number of mature synaptic contacts in the “Hypoxia” group was significantly lower than that in the “Sham” group (“Hypoxia” 20.33 ± 12.14%, *p* < 0.05, ANOVA) (Figure 2).

GDNF maintained the synaptic apparatus structure in hippocampal cells. The percentage of mature synapses and the structure of the synaptic pool in the “Hypoxia+GDNF” group were not different from those in the “Sham” group (Figure 2).

Numerous cells with destroyed organelles were visualized in primary hippocampal cultures one day after hypoxia modelling. In particular, the number of mitochondrial cristae in a neuron body was significantly decreased in the “Hypoxia” group compared to that in the “Sham” group, and their conformation was modified. In the neuronal outgrowth, osmiophilic mitochondria with altered shapes or complete destroyed organelles were detected, indicating deficiencies in the cellular energy supply (Figures 1(d)–1(f)). Osmiophilic synaptic bubbles with a double membrane were visualized among synaptic vesicles. Ultrastructural changes were detected in both neurons and glial cells. In the fields of view, we often detected “empty” glial outgrowths in which mitochondria with different defects were sometimes encountered. In most cases, the organelles were osmiophilic and had a round shape and extended cristae. In several cases, mitochondria with completely destroyed internal membranes were also detected (Figure 1(e)).

Significant changes in the ultrastructure of the mitochondrial apparatus were observed in the “Hypoxia+GDNF” cultures. In most cases, deformed mitochondria were visualized in the neuron body (Figures 1(g)–1(i)). The ultrastructures of mitochondria in small dendrites and neuronal outgrowths in the “Hypoxia+GDNF” group did not differ from those in the “Sham” group. Therefore, the neuroprotective action of GDNF is associated with preservation of the synaptic apparatus and part of the mitochondrial apparatus localized to neuronal outgrowths.

**Figure 3:**
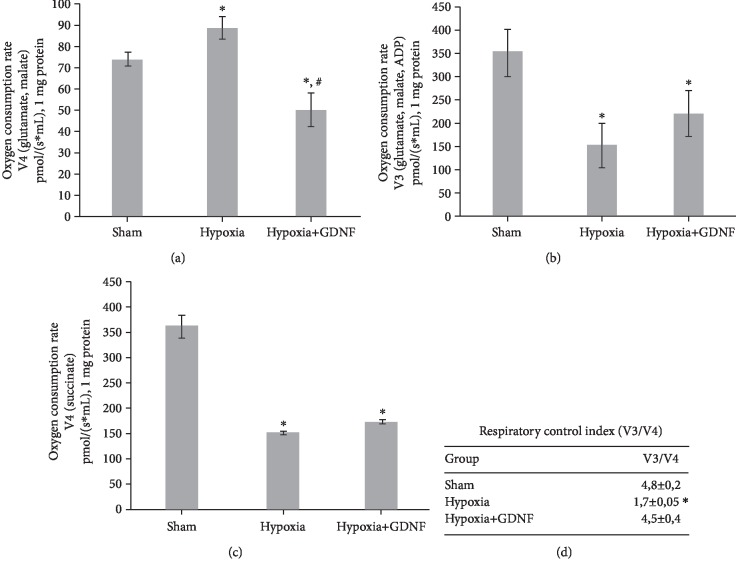
Mitochondrial functional activity in primary hippocampal cell cultures one day after acute normobaric hypoxia modelling *in vitro*. (a) The rate of oxygen consumption by mitochondria in the V4 state (glutamate and malate substrates). (b) The rate of oxygen consumption by mitochondria in the V3 state (glutamate and malate substrates, ADP). (c) The rate of oxygen consumption by mitochondria in the V4 state (succinate substrate). (d) The degree of mitochondrial respiratory chain coupling (V3/V4 ratio). The data represent the mean values ± SEMs from three independent experiments. Statistical significance was calculated by one-way ANOVA; *p* < 0.05; ^∗^versus “Sham”; ^#^versus “Hypoxia”.

Studies on the functional state of mitochondria confirmed the ultrastructural data analysis. We showed that GDNF reduces the hypoxia-induced hyperactivation of mitochondrial respiratory chain complexes I and II. GDNF reduced the pathologically increased rate of oxygen consumption by mitochondria in oxidation of glutamate and malate (substrates of complex I) by 43.5% relative to the “Hypoxia” group (V4 state, pmol/(s∗ml), 1 mg protein: “Sham” 73.99 ± 2.26, “Hypoxia” 88.8 ± 5.3, and “Hypoxia+GDNF” 50.2 ± 7.9). On the other hand, GDNF decreased the values of V4 state compared to the “Sham” group by 32% (Figure 3(a)). At the same time, the use of GDNF did not affect the oxidative phosphorylation rate (Figure 3(b)) and V4 state (succinate substrate oxidation) (Figure 3(c)), as the values of the “Hypoxia” and “Hypoxia+GDNF” groups did not differ significantly.

Interestingly, GDNF showed its neuroprotective action in maintaining the respiratory control index values (Figure 3(d)). Compared to the “Sham” group, hypoxia decreased the ratio of V3 and V4 by 2.2 times, whereas in the “Hypoxia+GDNF” group, this parameter did not differ significantly (V3/V4: “Sham” 3.8 ± 0.2, “Hypoxia” 1.7 ± 0.05, and “Hypoxia+GDNF” 4.5 ± 0.4).

**Figure 4:**
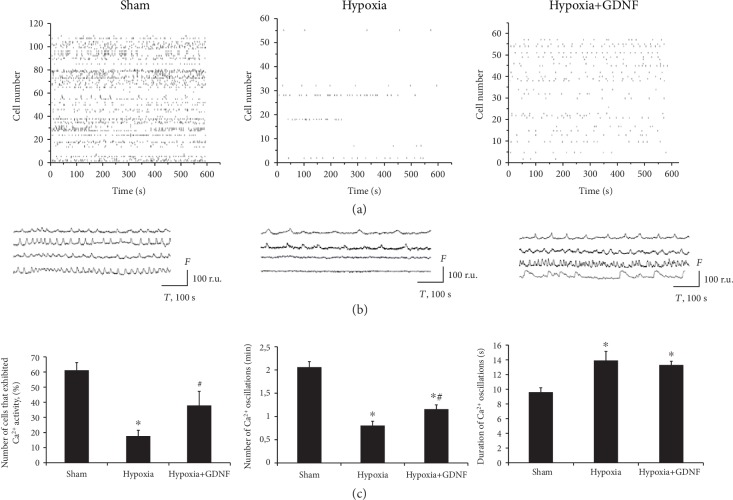
Features of spontaneous calcium activity in primary hippocampal cultures on day 7 of the posthypoxic period. (a) Raster diagrams of spontaneous Ca^2+^ activity. The moments of oscillations occurrence are presented as strokes. (b) Representative recordings of spontaneous Ca2+ oscillations,. F: fluorescence intensity (relative units (r.u.)); T: time (seconds). (c) Main parameters of spontaneous calcium activity in primary hippocampal cultures: (left) proportion of cells exhibiting calcium activity; (middle) number of Ca^2+^ oscillations per min; (right) duration of Ca^2+^ oscillations. The data represent the mean values ± SEMs from three independent experiments. Statistical significance was calculated by one-way ANOVA; *p* < 0.05; ^∗^versus “Sham”; ^#^versus “Hypoxia”.

Next, we investigated the features of functional calcium activity in primary hippocampal cultures in the posthypoxic period. Analysis of the spontaneous functional activity and morphology of neuron-glial networks in culture is considered an effective approach for studying the dynamics, structure, and intercellular communications in neuron-glial networks. Primary hippocampal cell cultures generate stereotypical patterns of activity, which are characterized by the synchronized activity of numerous cells [24, 25, 31]. High-resolution fluorescence calcium imaging allows for the analysis of network activity dynamics under different influences, including hypoxic injury. A stable pattern of calcium activity was observed by DIV 14 (number of cells exhibiting Ca^2+^ activity: 61.09 ± 5.01%; frequency of Ca^2+^ oscillations: 2.06 ± 0.12 osc/min; and duration of Ca^2+^ oscillations: 9.69 ± 0.47 s). In addition, a raster diagram of spontaneous Ca^2+^ activity allows for the selection of regular synchronous events associated with synaptic transmission and a consolidated response in the neural network. Destructive processes in functional Ca^2+^ activity were highly marked in the posthypoxic period (Figure 4). The main parameters of spontaneous Ca^2+^ activity in the “Hypoxia” group were significantly lower than those in the “Sham” group (“Hypoxia”: number of cells exhibiting Ca^2+^ activity: 17.5 ± 3.91%; frequency of Ca^2+^ oscillations: 0.81 ± 0.09 osc/min; and duration of Ca^2+^ oscillations: 13.96 ± 1.12 s). Moreover, hypoxia led to total disruption of the network structure.

GDNF partially abolished the consequences of hypoxia on the functional level. In the “Hypoxia+GDNF” group, the number of cells exhibiting Ca^2+^ activity and the frequency of Ca^2+^ oscillations were significantly higher than those in the “Hypoxia” group (Hypoxia+GDNF: number of cells exhibiting Ca^2+^ activity: 37.65 ± 9.55%; frequency of Ca^2+^ oscillations: 1.17 ± 0.08 osc/min; and duration of Ca^2+^ oscillations: 13.33 ± 0.42 s). Analysis of the functional activity pattern revealed the preservation of regular synchronous events, indicating the maintenance of neuron-glial network functional activity under hypoxia.

These data are consistent with the ultrastructural analysis, indicating that GDNF preserves synaptic contacts, which reportedly ensure the maintenance of the functional activity of cells participating in the formation of spontaneous Ca^2+^ activity.

**Figure 5:**
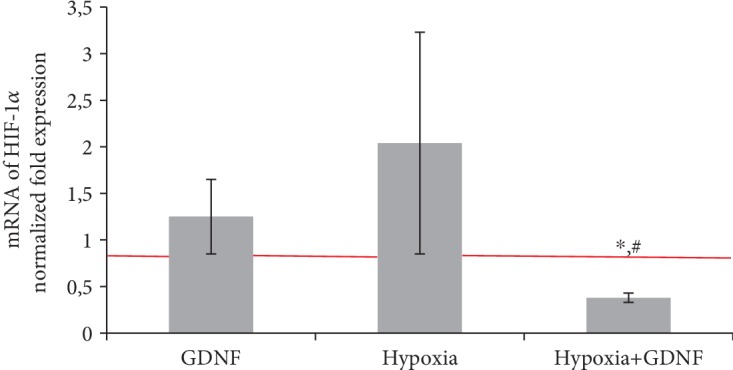
Features of HIF-1*α* transcription factor gene expression on day 1 of the posthypoxic period. Data are normalized to the reference gene (Oaz1). The data represent the mean values ± SEMs from three independent experiments. Statistical significance was calculated by one-way ANOVA; *p* < 0.05; ^∗^versus “Sham”; ^#^versus “Hypoxia”.

Modelled hypoxia significantly increased HIF-1*α* expression, indicating the adequacy of the selected hypoxia model and confirming a general biological pattern of adaptive response development in primary hippocampal culture cells. Pretreatment with GDNF (1 ng/ml) led to a significant decrease in HIF-1*α* expression, which could be considered a molecular mechanism of GDNF action (Figure 5).

We analysed the molecular mechanisms most likely involved in the positive effects of GDNF. To determine which intracellular pathways mediate the survival effects of GDNF on neuronal cells, we investigated the contributions of the main component of the GDNF receptor, RET kinase, to GDNF neuroprotective properties and key enzymes, including the Ras/МАРK, PI3K/Akt, and Jak/STAT pathways (RAF, MAP2K1/2, Erk2, AKT1, Jak1, and Jak2). In normoxia-only RET, RAF kinase inhibition significantly changed the viability of primary hippocampal cells (Table 1).

However, in the modelled hypoxic state, the use of the main GDNF metabolic cascade inhibitors led to a sharp decrease in cell viability. RET kinase inhibition had the most pronounced effect since RET kinase activity is crucial for GFR-mediated GDNF action (Table 2).

Analysis of cell viability in the posthypoxic period in the context of combined application of GDNF and kinase inhibitors showed that the neuroprotective effect of this neurotrophic factor was mainly associated with RET, AKT1, Jak1, and Jak2 kinase activities. Thus, the role of the PI3K/Akt and Jak/STAT metabolic pathways in the neuroprotective effect of GDNF was revealed.

**Figure 6:**
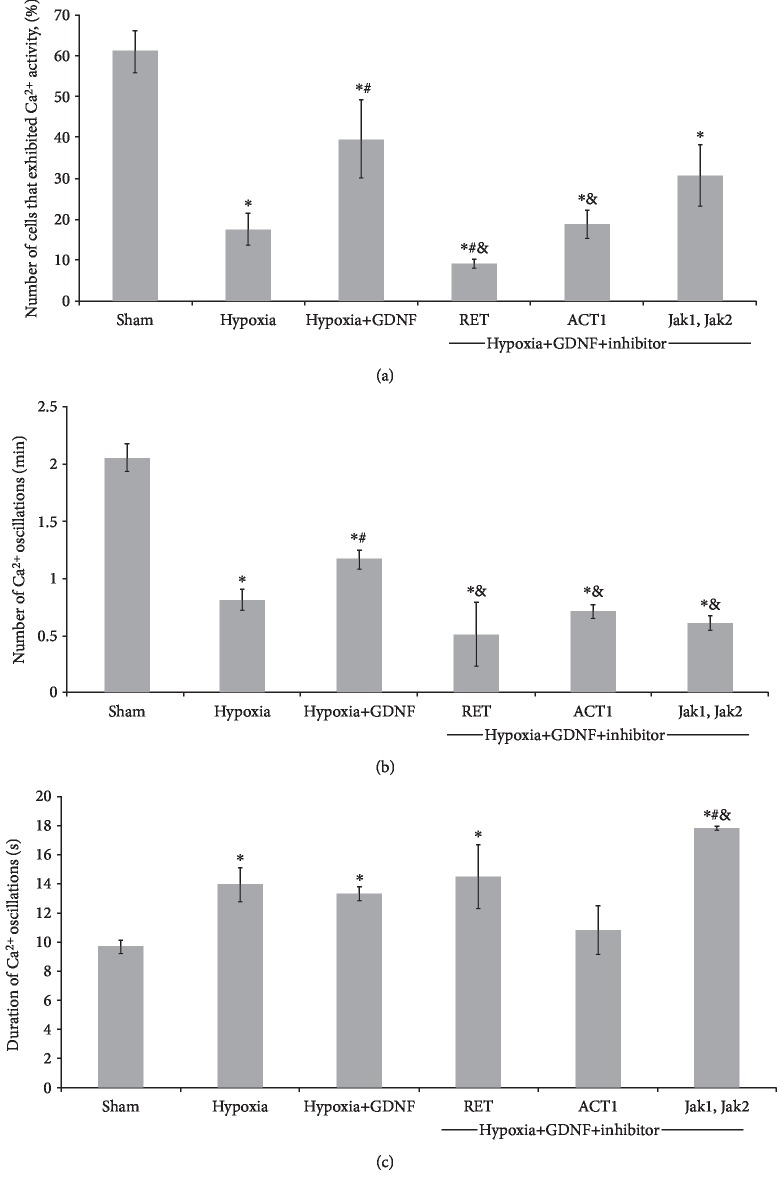
Main parameters of spontaneous calcium activity in primary hippocampal cultures on day 7 of the posthypoxic period under the inhibition of intracellular kinases: (a) proportion of cells exhibiting calcium activity; (b) number of Ca2+ oscillations per min; and (c) duration of Ca2+ oscillations. The data represent the mean values ± SEMs from three independent experiments. Statistical significance was calculated by one-way ANOVA; *p* < 0.05; ^∗^versus “Sham”; ^#^versus “Hypoxia”; ^&^versus “Hypoxia+GDNF”.

Next, we investigated the influence of these kinase blockers on the metabolic activity of primary hippocampal cultures. A study on the influence of selected metabolic cascades on the functional activity of primary hippocampal cells revealed that changes in the main functional parameters of Ca^2+^ activity were associated with cell viability (Figure 6). Compared with the application of hypoxia alone, the application of a RET kinase inhibitor completely abolished the neuroprotective effect of GDNF on Ca^2+^ network activity and significantly decreased the number of cells exhibiting Ca^2+^ events. The inhibition of AKT1 kinase, which is included in the PI3K/Akt metabolic cascades, also eliminated the neuroprotective effect of GDNF. Jak/STAT metabolic pathway inhibition had a less pronounced effect; however, compared with hypoxia and GDNF, it reduced the frequency of calcium oscillations.

**Figure 7:**
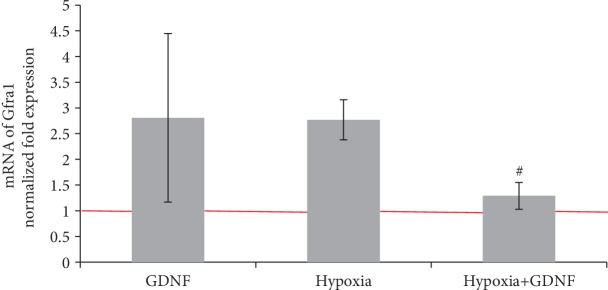
Features of Gfra1 gene expression encoding the GFR*α* receptor on day 1 of the posthypoxic period. Data are normalized to the reference gene (Oaz1) and represent the mean values ± SEMs from three independent experiments. Statistical significance was calculated by one-way ANOVA; *p* < 0.05; ^∗^versus “Sham”; ^#^versus “Hypoxia”.

Analysis revealed that the neuroprotective effect of GDNF was associated with activation of the GRF receptor and dimerization of RET kinase membrane plots. Using real-time PCR, we analysed the level of GFR*α*1 expression in the hypoxic state and under GDNF application. Pretreatment with GDNF in hypoxia modelling significantly decreased the expression of the GFR*α*1 receptor (Figure 7). This decreased expression can be considered a compensatory response to the increased neurotrophic factor concentration in the blood and indirectly indicates that the concentration we used can be reduced without the loss of GDNF neuroprotective properties.

## 4. Discussion

GDNF is an important signalling molecule that plays an essential role in promoting cellular survival in the mature brain [32]. Numerous experimental studies have revealed that GDNF has pronounced neuroprotective properties, including its ability to protect nervous tissue under hypoxic states [13, 33] and in neurodegenerative diseases [34–37]. These data suggest that molecular and cellular mechanisms activated by GDNF have an extremely high adaptive potential. On the other hand, some data exist showing that GNDF can activate pathological processes over a long-term period [38]. Therefore, there is a need not only to study the positive effects of GDNF but also to identify the specific molecular mechanisms whose activation mediates those effects. However, previous studies have not analysed the influence of GDNF on functional neural network activity and synaptic structure or investigated the related molecular mechanisms.

The present study showed the antihypoxic and neuroprotective effects of GDNF on primary hippocampal cultures in modelled acute hypoxic conditions. Our data suggest that an acute hypoxic episode induces irreversible changes in the ultrastructure of neurons and astrocytes in primary hippocampal cultures and alters the functional calcium activity of neuron-glial networks. Destructive changes in the mitochondrial apparatus were revealed 24 hours after hypoxia modelling. Moreover, changes in mitochondrial functional activity, including an increase in the basal oxygen consumption rate and mitochondrial respiratory chain complex II activation, were observed in the context of decreased stimulated respiration intensity. The results showing that GDNF contributes to preservation of the mitochondrial structure, mainly in neuronal outgrowths, are of particular interest. Undoubtedly, neural regulation and neurons themselves constitute one of the most complex bodily systems in humans. The modern concept of brain functional activity suggests that the neural network is the minimal functional unit of the nervous system [39]. However, a single neuron is extremely heterogeneous and is not a simple element. Reinforcing the glucose-lactate shuttling concept suggests that the neuron cannot be considered a metabolically homogeneous cell [40, 41]. At the cellular level, substantial distances between a neuron body and distal endings of neuronal outgrowth as well as extremely intensive energy exchanges in synaptic clefts conditionally divide the neuron into two parts. The first one is a metabolically calm cell body that never divides whose main function is to regulate and maintain organelle integrity and the synthesis of proteins, which are delivered to the synaptic endings by axonal transport. The second part is areas of neuronal outgrowth, wherein the most energy-intensive processes in human organisms take place, consuming 25% of oxygen and 20% of all energy absorbed from food. GDNF is presumably capable of triggering unknown cellular mechanisms that contribute to the protection of mitochondria in synaptic processes, the most important regions for neurons, wherein mitochondria in the neuron body can be disrupted more than those under hypoxic conditions. This phenomenon was confirmed by an integrated study of mitochondrial functional activity. However, GDNF-mediated preservation of the mitochondrial pool in synaptic contacts and distal areas of neuronal outgrowth has a positive systemic effect and certainly requires further investigation.

Interestingly, hypoxia significantly increased the basal oxygen consumption rate and mitochondrial respiratory chain complex II activity, as the stimulated respiration of both complexes was decreased (Figure 3). The increased activity of respiratory chain complexes I and II could be considered a compensatory reaction. However, a decrease in the response of mitochondria to selective biochemical stimuli indicates defective metabolic regulation processes and dissociation of oxidative phosphorylation coupled with activation of free radical processes. The decrease of respiratory control index values to 1 confirms the separation of oxidation and phosphorylation processes in mitochondria during their damage in the posthypoxic period [42].

On the one hand, pretreatment of GDNF (1 ng/ml) decreased the pathologically increased rate of oxygen consumption by mitochondria in oxidation of glutamate and malate complex I substrates (V4 state) in hypoxia but did not support oxidative phosphorylation values and V4 state (succinate substrate oxidation). On the other hand, GDNF application maintained the respiratory control index (V3/V4 ratio), reflecting the degree of mitochondrial respiratory chain coupling.

Our data showed that GDNF has a protective effect on the calcium activity of neuron-glial networks characterized by maintenance of the main parameters of Ca^2+^ activity and network functional structure. In contrast, hypoxia led to total disruption of the network structure. Thus, network disruption and loss of synchronization between individual network elements are among the most devastating consequences of hypoxia. This is related to the destruction of synaptic contacts and death of the part of cells included in a neuron-glial network. Pretreatment of GDNF (1 ng/ml) allows maintaining the number of cells exhibiting calcium activity and the frequency of calcium oscillations at significantly higher level than in the “Hypoxia” group. The data revealed that the neuroprotective effect of GDNF is related to the preservation of network activity and ultrastructure of synaptic contacts.

Notably, a significant increase in the duration of Ca^2+^ activity occurs under hypoxic conditions, potentially for two reasons. First, because astrocytes are more resistant to hypoxia [43–45], these cells mainly exhibit functional activity characterized by slower Ca^2+^ dynamics and prolonged Ca^2+^ oscillations in cultures subjected to oxygen deficiency [46–48].

Second, increasing the duration of Ca^2+^ oscillations could be associated with Ca^2+^ increases in the cytoplasm as a result of excitotoxicity development after hypoxia [49–51]. These processes lead to an increased calcium ion concentration in the cytoplasm and saturation of Ca^2+^-binding systems in a cell.

Current data on ultrastructural and functional disruption of mitochondria and the increased duration of Ca^2+^ oscillations after acute normobaric hypoxia are consistent with published data indicating that oxygen and glucose deprivation can lead to an iGluR-dependent increase in the extracellular glutamate concentration (excitotoxicity), glutamate transporter activation, and degradation of mitochondria in astrocytic [20] and neuronal outgrowths [21, 52, 53]. Mitochondria play an important role in the development of dysfunction in all cell types during ischaemia; however, neurons are the most sensitive. As a result of ischaemic oxidative stress, increased Ca^2+^ concentrations and reactive oxygen disturb mitochondrial membrane permeability, and the mitochondrial permeability transition pore is formed, which ensures a free flow of dissolved low-molecular substances. Mitochondria swelling and disruption of the outer membrane lead to proapoptotic factor penetration into the cytoplasm (cytochrome C and apoptosis-inducing factor), which are normally sequestered and maintained in an inactivated state in the space between the inner and outer mitochondrial membranes [54]. Our electron microscopy data on the ultrastructure of primary hippocampal cells in the posthypoxic period confirm this hypothesis.

According to another hypothesis, enhanced oxidation of glutamate penetrating the cell through its reuptake from the extracellular space could stimulate the loss of mitochondria due to increase reactive oxygen species (ROS) levels [20]. Glutamate participates in the tricarboxylic acid cycle by conversion to *α*-ketoglutarate [55]. *α*-Ketoglutarate is rearranged to succinyl-CoA by the *α*-ketoglutarate dehydrogenase complex and generates 2-3 times more ROS than other dehydrogenases in the Krebs cycle [56, 57].

Thus, our data demonstrate that the increased duration of Ca^2+^ oscillations in the posthypoxic period may be the reason for mitochondrial functional activity defectiveness and organelle disruption. This phenomenon is consistent with electron microscopy data analysis indicating that pretreatment with GDNF preserves synaptic contacts and the structure of mitochondria in neuronal outgrowth, probably due to partial stabilization of the functional Ca^2+^ activity in cells.

The activation of HIF is one of the key adaptive mechanisms in the hypoxic state. The transcription factor HIF-1 controls oxygen transfer to tissues and adapts cells to oxygen deficiency by regulating the expression of gene products involved in cell energy metabolism, glucose transport, apoptosis, erythropoiesis, angiogenesis, and proliferation regulation [58]. However, data on the role of HIF-1*α* in brain adaptation to oxygen deficiency are contradictory. Despite studies demonstrating that HIF-1*α* stimulates erythropoiesis and angiogenesis, which increase an organism's resistance to hypoxia [59–62], several studies indicate a negative effect of HIF-1*α* on nervous system cell adaptation to hypoxic injury [63, 64]. HIF-1*α* is an activator of the proapoptotic gene р53 in a hypoxic state [65, 66] and in response to head trauma [67]. Thus, the contributions of the transcription factor HIF-1*α* to the neuroprotective effect of GDNF is of special interest. The current study also revealed the role of GDNF in the regulation of HIF-1*α* transcription factor expression under hypoxic conditions.

**Figure 8:**
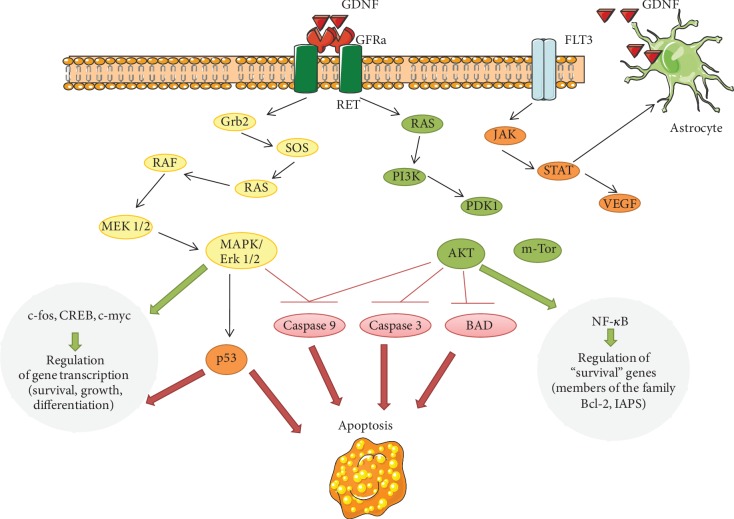
Glial cell line-derived neurotrophic factor signalling pathways. GDNF binds to the GFR*α*/Ret-receptor complex and activates phosphoinositide-3 kinase (PI3K) and mitogen-activated protein kinase (MAPK) pathways. Akt: protein kinase В; BAD: proapoptotic protein; c-fos: protein-regulator of transcription of a number of inducible genes; c-myc: gene, encoding protein-transcription factor; CREB: cAMP-dependent transcription factor; GDNF: glial cell line-derived neurotrophic factor; GFR*α*: specific coreceptor for GDNF; Grb2: adaptor protein; FLT3: receptor-type tyrosine-protein kinase FLT3; Jak: Janus kinase, intracellular, nonreceptor tyrosine kinase; m-TOR: protein kinase with serine-threonine specificity; MAPК: mitogen-activated protein kinase; МЕК: kinase of MAPK; NF*κ*B: transcription nuclear factor kappa В; p53: transcription factor regulating the cellular cycle; PDK1: phosphoinositide independent protein kinase; PI3K: phosphoinositol-3-kinase; Raf: serine-threonine protein kinase; Ras: small GTF-binding protein; Ret: receptor with tyrosine kinase activity; SOS: guanine nucleotides exchange factor; STAT: signal transducer and activator of transcription proteins; VEGF: vascular endothelial growth factor.

GDNF is currently known to activate several intracellular signalling pathways and kinases (Figure 8). The Ras/МАРK and PI3K/Akt cascades can be activated when GDNF binds to its main receptor, which consists of the ligand-binding component GFR*α* and the tyrosine kinase RET (second component of the receptor complex) [13, 14, 68–70]. Together, these components form a functional receptor block for GDNF binding [14]. Since inhibition of Act1 kinase negates the protective effect of GDNF on the viability of cells in a hypoxic state, we can argue that the PI3K/Akt cascade plays a leading role in the implementation of these effects. These data are consistent with several studies on the ability of GDNF to maintain mitochondrial activity. Using a mouse model of Parkinson's disease, Meka et al. showed that GDNF improves impaired mitochondrial function by activating the NF-*κ*B transcription factor, mediated by RET kinase through the phosphoinositide-3-kinase (PI3K) pathway [71].

Data on the role of the Jak kinase-mediated action of GNDF are of special interest. While the Jak/STAT pathway is not associated with GDNF receptor activation, several studies have shown that its activation can regulate GDNF production in glial cells [72–74]. Thus, conducting studies on the possible contribution of this metabolic cascade to the neuroprotective effect of GDNF is interesting. A blockade of Jak kinase may decrease the endogenous production of GDNF, which could be an exciting area for upcoming research.

Thus, our data suggest that an acute hypoxic episode induces irreversible changes in the ultrastructure of neurons and astrocytes in primary hippocampal cultures and in the functional calcium activity of neuron-glial networks. Moreover, changes in mitochondrial functional activity, including an increase in the basal oxygen consumption rate and mitochondrial respiratory chain complex II activation, were observed in the context of decreased stimulated respiration intensity. GDNF application protects nerve cells from hypoxic damage and maintains cell viability. The neuroprotective effect of GDNF is associated with the preservation of spontaneous Ca^2+^ network activity, the structure of the synaptic apparatus, and the number of mature chemical synapses. Interestingly, the use of GDNF preserved the normal structure of mitochondria only in neuronal outgrowths and not in the soma.

The antihypoxic and neuroprotective properties of GDNF are realized through GFR*α* receptor activation. RET kinase, a component of the receptor complex, plays a key role in the protective effect of GDNF. Moreover, a crucial role of the PI3K/Akt pathway in the development of neuroprotective effects related to GFR*α* receptor complex activation has been revealed. In addition, we propose that the neuroprotective effect of this neurotrophic factor is associated with the regulation of transcription factor HIF-1*α* expression.

The results obtained herein indicate the need to study the effect of GDNF on the molecular mechanisms of mitochondrial stability maintenance in oxygen-deficient conditions and highlight the need to investigate previously unexplored molecular cascades, mediating a high adaptive potential of GDNF.

## Figures and Tables

**Table 1:** Analysis of cell viability in primary hippocampal cultures under the inhibition of intracellular kinases in normoxic conditions.

	Number of viable cells day one after inhibitor application (%)	Number of viable cells on day 7 after inhibitor application (%)
Sham	96.78 ± 0.95	97.13 ± 2.24
RET kinase inhibitor	89.63 ± 2.69^∗^	86.06 ± 1.61^∗^
RAF kinase inhibitor	96.24 ± 1.08	90.38 ± 1.48^∗^
MAP2K1/2, Erk2, and RAF kinase inhibitors	93.68 ± 1.03	94.35 ± 0.76
AKT1 kinase inhibitor	98.22 ± 0.28	96.84 ± 0.52
Jak1 and Jak2 kinase inhibitors	94.12 ± 1.32	95.09 ± 2.12

The data represent the mean values ± SEM from six independent experiments. Statistical significance was calculated by one-way ANOVA; *p* < 0.05; ^∗^versus “Sham”.

**Table 2:** Analysis of cell viability in primary hippocampal cultures under the inhibition of intracellular kinases in hypoxia modelling.

Group	Number of viable cells on day 7 after inhibitor application (%)	Number of viable cells on day 7 after inhibitor and GDNF application (%)
Sham	92.28 ± 1.81	
Hypoxia	72.05 ± 5.73^∗^	
Hypoxia+GDNF	87.78 ± 4.63^∗#^	
Hypoxia+RET kinase inhibitor	30.03 ± 1.96^∗#^	28.27 ± 1.57^∗#&^
Hypoxia+RAF kinase inhibitor	74.48 ± 2.89^∗^	80.09 ± 2.18^∗^
Hypoxia+MAP2K1/2, Erk2, RAF kinase inhibitor	79.03 ± 3.64^∗^	81.21 ± 4.19^#^
Hypoxia+AKT1 kinase inhibitor	70.22 ± 3.61^∗^	77.96 ± 2.02^∗#&^
Hypoxia+Jak1, Jak2 kinase inhibitor	74.52 ± 3.61^∗^	75.22 ± 5.61^∗&^

The data represent the mean values ± SEMs from six independent experiments. Statistical significance was calculated by one-way ANOVA; ^∗^versus “Sham”; ^#^versus “Hypoxia”; ^&^versus “Hypoxia+GDNF”.

## Data Availability

The data used to support the findings of this study are available from the corresponding author upon request.
